# Succession in the caecal microbiota of developing broilers colonised by extended-spectrum β-lactamase-producing *Escherichia coli*

**DOI:** 10.1186/s42523-022-00199-4

**Published:** 2022-08-19

**Authors:** Ingrid Cárdenas-Rey, Teresita d. J. Bello Gonzalez, Jeanet van der Goot, Daniela Ceccarelli, Gerwin Bouwhuis, Danielle Schillemans, Stephanie D. Jurburg, Kees T. Veldman, J. Arjan G. M. de Visser, Michael S. M. Brouwer

**Affiliations:** 1 Department of Bacteriology, Host-Pathogen Interactions and Diagnostics Development, Wageningen Bioveterinary Research, Lelystad, The Netherlands; 2 Gezondheidscentrum Voor Pluimvee, Emmen, The Netherlands; 3 Department of Environmental Microbiology, Helmholtz Centre for Environmental Research - UFZ, Leipzig, Germany; 4 Institute of Biology, Leipzig University, Deutscher Platz 5e, 04103 Leipzig, Germany; 5 Laboratory of Genetics, Wageningen University & Research, Wageningen, The Netherlands; 6 Wageningen University & Research, Wageningen, The Netherlands

**Keywords:** Broilers, ESBL, *Escherichia coli*, Microbiota

## Abstract

**Background:**

Broilers are among the most common and dense poultry production systems, where antimicrobials have been used extensively to promote animal health and performance. The continuous usage of antimicrobials has contributed to the appearance of resistant bacteria, such as extended-spectrum β-lactamase-producing *Escherichia coli* (ESBL-Ec)*.* Here, we studied the ESBL-Ec prevalence and successional dynamics of the caecal microbiota of developing broilers in a commercial flock during their production life cycle (0–35 days). Broilers were categorised as ESBL-Ec colonised (ESBL-Ec^+^) or ESBL-Ec non-colonised (ESBL-Ec^−^) by selective culturing. Using 16S rRNA gene sequencing, we i. compared the richness, evenness and composition of the caecal microbiota of both broilers’ groups and ii. assessed the combined role of age and ESBL-Ec status on the broilers’ caecal microbiota.

**Results:**

From day two, we observed an increasing linear trend in the proportions of ESBL-Ec throughout the broilers' production life cycle, *X*^*2*^ (1, *N* = 12) = 28.4, *p* < 0.001. Over time, the caecal microbiota richness was consistently higher in ESBL-Ec^−^ broilers, but significant differences between both broilers’ groups were found exclusively on day three (Wilcoxon rank-sum test, *p* = 0.016). Bray–Curtis distance-based RDA (BC-dbRDA) showed no explanatory power of ESBL-Ec status, while age explained 14% of the compositional variation of the caecal microbiota, *F* (2, 66) = 6.47, *p* = 0.001.

**Conclusions:**

This study assessed the role of ESBL-Ec in the successional dynamics of the caecal microbiota in developing broilers and showed that the presence of ESBL-Ec is associated with mild but consistent reductions in alpha diversity and with transient bacterial compositional differences. We also reported the clonal spread of ESBL-Ec and pointed to the farm environment as a likely source for ESBLs.

**Supplementary Information:**

The online version contains supplementary material available at 10.1186/s42523-022-00199-4.

## Background

Poultry are considered one of the most important animal protein sources and are a major driver of the global growth in protein production [1] but at the same time, the poultry industry has been identified as an important source of antimicrobial resistance (AMR) [2]. Broiler chicken farms are among the most common and dense poultry production systems, where antimicrobials have been used extensively to promote animal health and performance. The broad and continuous therapeutic and prophylactic usage of antimicrobials has contributed to the global emergence of extended-spectrum beta-lactamases (ESBLs) among pathogenic and commensal *Enterobacterales*, especially *Escherichia coli* [3, 4].

ESBL-producing *Enterobacterales* have been recognised as a public health threat due to their capacity to inactivate a large group of critically important antibiotics belonging to the group of β-lactams, like the extended-spectrum cephalosporins (ESC) [5, 6]. Despite the crucial action plans carried out by several European countries against AMR, e.g. the ban of growth promoters in The Netherlands since 2006 and the changes in policies for antimicrobial usage in animals [7, 8], the spread of commensal ESBL-producing *Enterobacterales* is still observed across animal production systems. This spread has been associated with the horizontal gene transfer (HGT) of ESBL families like TEM, CTX-M and SHV through conjugative plasmids capable of persisting successfully in the host and the environment [9].

Alternatives to reduce ESBL HGT in poultry productions include modulating nutrient-rich and densely populated microbial ecosystems like the broilers’ caeca [10]. The caeca are finger-like shaped organs that harbors the highest number of microorganisms in the broiler’s gastrointestinal tract, which facilitates the exchange of ESBL plasmids among commensal and pathogenic bacteria [11]. The development of the caecal microbiota of commercial broilers has been associated primarily with environmental sources [12, 13]. Immediately after hatching, chickens are colonised by exogenous microorganisms that can potentially act as a reservoir of ESBL genes in the caecal microbiota, including *E. coli* [14, 15]. As the broiler ages, the caecal microbiota undergoes successional changes, which involve rapid and directional turnover towards a more diverse community with a lower abundance of *Enterobacterales,* including *E. coli* [16, 17]. How the timing of colonisation of ESBL*-*producing *E. coli* (ESBL-Ec) is affected by these microbial successional dynamics, and whether the presence of ESBL-Ec affects the development of the caecal microbiota of broilers is unknown.

Here, we studied the ESBL-Ec prevalence and successional dynamics of the caecal microbiota of developing broilers in a commercial flock during their production life cycle. Broilers were categorised as ESBL-Ec colonised (ESBL-Ec^+^) or ESBL-Ec non-colonised (ESBL-Ec^−^) by selective culturing. Using 16S rRNA gene sequencing, we compared the richness, evenness and composition of the caecal microbiota of both broilers’ groups and assessed the combined role of age and ESBL-Ec status on the microbiota. We observed a linear increasing trend in the proportions of ESBL-Ec throughout the broilers' production round. Over time, microbial richness was consistently higher in ESBL-Ec^−^ broilers, but significant differences between groups were found exclusively on day three. Bray–Curtis distance-based RDA (BC-dbRDA) showed no explanatory power of ESBL-Ec status, while age explained 14% of the compositional variation of the caecal microbiota.

## Results

### Prevalence and typing of ESBL producing *E. coli*

**Fig. 1:**
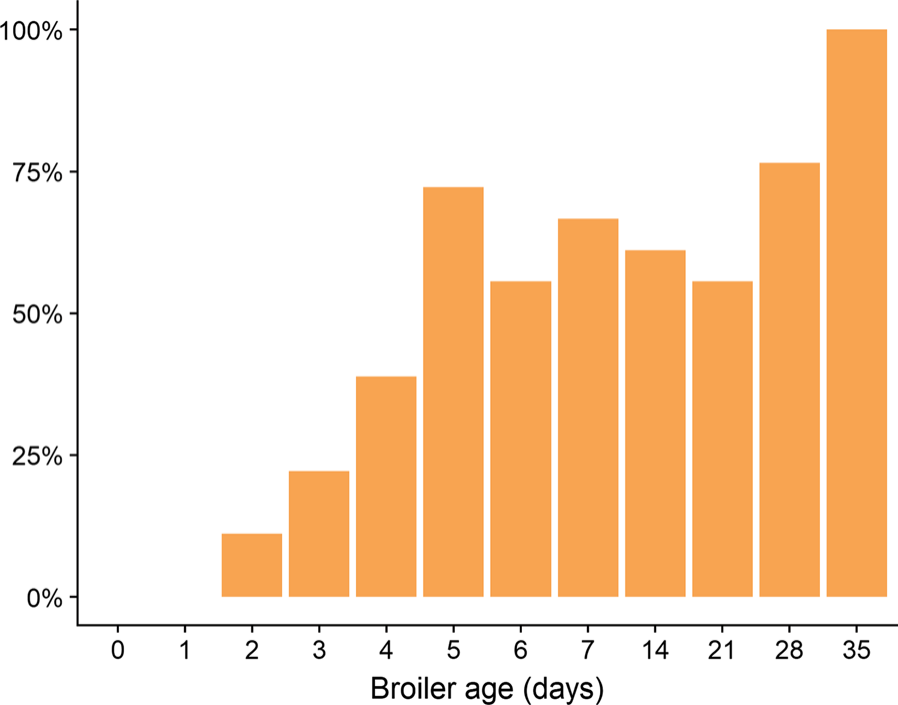
ESBL*-E.coli* prevalence throughout the broilers’ life cycle. Trends of ESBL-Ec prevalence in the total broiler population

Caecal samples were collected on a Dutch commercial broiler farm throughout the production round (days 0–7, 14, 21, 28 and 35). Culture-based methods were applied to each caecal sample to discriminate between ESBL-Ec^+^ and ESBL-Ec^−^ broilers. ESBL-Ec were detected from day 2 with a sample prevalence of 11% CI [1; 34] and rapidly increased to 72% CI [46; 90] until day 5. Prevalence fluctuated between days 6 and 28 but remained above 50%. All caecal samples were positive for ESBL-Ec on day 35 (Fig. 1). An increasing linear trend in the proportions of ESBL-Ec was observed throughout the broilers' production round, *X*^*2*^ (1, *N* = 12) = 28.4, *p* < 0.001.

The most prevalent ESBL/AmpC-gene families responsible for the ESC-resistant phenotype in The Netherlands (CTX-M, SHV, TEM and CMY) were determined using real-time PCR. Both *bla*_SHV_ and *bla*_TEM_ PCR products were amplified for all 109 ESC-resistant *E. coli* isolates. Sanger sequencing of 18 of these products spread over the production round indicated that all strains carried the ESBL gene *bla*_SHV-12_ and the non-ESBL beta-lactamase *bla*_TEM-1b_ (see Additional file 1). Bacterial transformation and subsequent PCR-based replicon typing (PBRT) demonstrated that *bla*_SHV-12_ genes were located on IncI1α plasmids in all 18 isolates. From these, 10 isolates spread over the production round were chosen for in-depth analysis. Antibiotic susceptibility testing (EUVSEC3) showed identical antimicrobial resistance profiles in all 10 isolates (see Additional file 2). PCR and Sanger sequencing showed that all 10 isolates belonged to the multilocus sequence typing (MLST) type 1011, while all IncI1α plasmids belonged to pMLST type 3.

### Characterisation of the caecal bacterial composition of ESBL-Ec^+^ and ESBL-Ec^−^ broilers

Due to the rapid increase of ESBL-Ec prevalence over time, microbiota comparisons between ESBL-Ec^+^ and ESBL-Ec^−^ broilers were only possible between days 3 and 28. Except for two phyla, no significant differences in the relative abundances of phyla were found between broilers' groups. *Bacteroidetes* were significantly higher in ESBL-Ec^+^ broilers on day 21 (ANCOM-BC, *p* = 0.003), while *Tenericutes* were significantly higher in ESBL-Ec^−^ broilers on day 28 (ANCOM-BC, *p* < 0.001). Both phyla represented only a small part of the total community (< 5%).

**Fig. 2:**
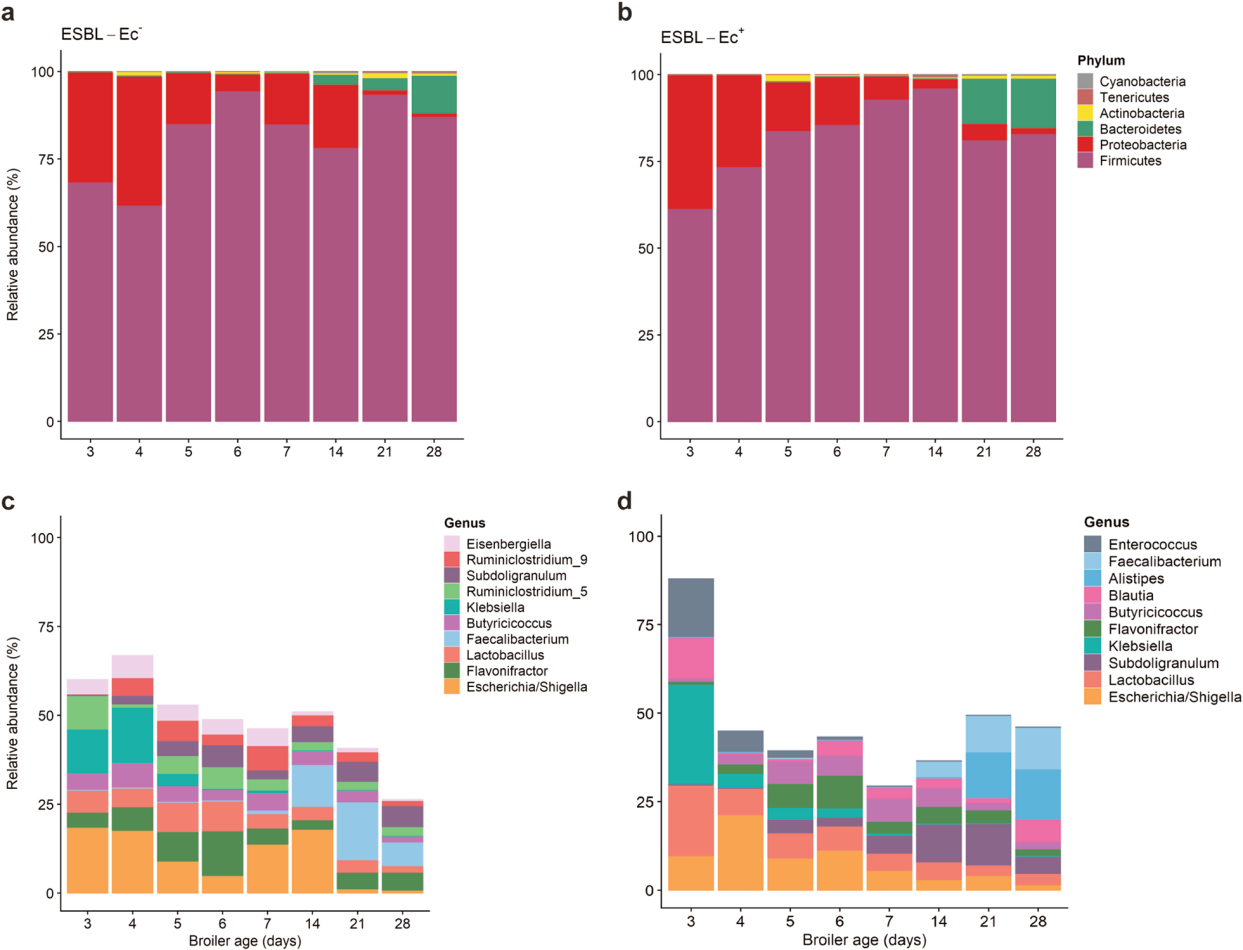
Caecal microbiota composition of ESBL-Ec^−^ and ESBL-Ec^+^ broilers. **a**–**b** Relative abundance of bacterial phyla of ESBL-Ec^−^ and ESBL-Ec^+^ broilers from day 3–28. **c**–**d** Relative abundance of the top ten genera of ESBL-Ec^−^ and ESBL-Ec^+^ broilers. The relative abundance of each phylum and genus is shown in decreasing order from bottom to top

Of the six bacterial phyla identified, *Firmicutes* were the most abundant with an average relative abundance of 77.9% ± 20.3, followed by *Proteobacteria* (18.2% ± 22.0) and *Bacteroidetes* (3.1% ± 6.3)*.* Across all samples, *Actinobacteria, Tenericutes* and *Cyanobacteria* combined represented only 0.8% ± 0.2 of the total taxa. *Firmicutes and Proteobacteria* predominated in the developing caecal microbiota of ESBL-Ec^−^ and ESBL-Ec^+^ broilers until day 14 (Fig. 2a,b). While the relative abundances of these phyla fluctuated during this period, no significant differences over time were found (ANCOM-BC, *p* > 0.05). *Bacteroidetes* were detected from day 14 onwards with a continuous increase in numbers until the end of the broiler's production life (16.6% ± 5.1).

Shifts in relative abundances of families were observed over time (see Additional file 3). However, no differential abundance was found between ESBL-Ec^+^ and ESBL-Ec^−^ broilers (ANCOM-BC, *p* > 0.05). Within the phylum *Firmicutes,* families *Ruminococcaceae* and *Lachnospiraceae* were the most prevalent over time in both broilers’ groups. *Proteobacteria* and *Bacteroidetes* were represented by the families *Enterobacteriaceae* (99%) and *Rikenellaceae* (100%), respectively. The main representative families of *Actinobacteria* were *Eggerthellaceae* and *Bifidobacteriaceae,* while for *Tenericutes,* it was *Anaeroplasmataceae.*

The ten most abundant genera in both broiler groups made up 45.7% ± 1.4 and 52.9% ± 2.7 of the total microbial community of ESBL-Ec^+^ and ESBL-Ec^−^ broilers, respectively. Only on day 3, the bacterial community of ESBL-Ec^+^ broilers was dominated by five genera; *Klebsiella, Lactobacillus, Enterococcus, Blautia* and *Escherichia/Shigella* (87% of the whole community)*,* while in ESBL-Ec^−^ broilers, the bacterial composition was much more diverse (Fig. 2c, d). The relative abundances of *Eisenbergiella* and *Subdoligranulum* were significantly higher in ESBL-Ec^−^ broilers on days 3 and 4, respectively (ANCOM-BC, *p* < 0.001). *Alistipes* was the most abundant taxon in ESBL-Ec^+^ broilers on days 21 (13.1% ± 5.5) and 28 (14.5% ± 5.4). Significant differences were found only on day 21 where in ESBL-Ec^−^ broilers* Alistipes* made up only 3.8% ± 2.2 of the community (ANCOM-BC, *p* = 0.01).

### Relative abundance of *Escherichia*/*Shigella*

**Fig. 3:**
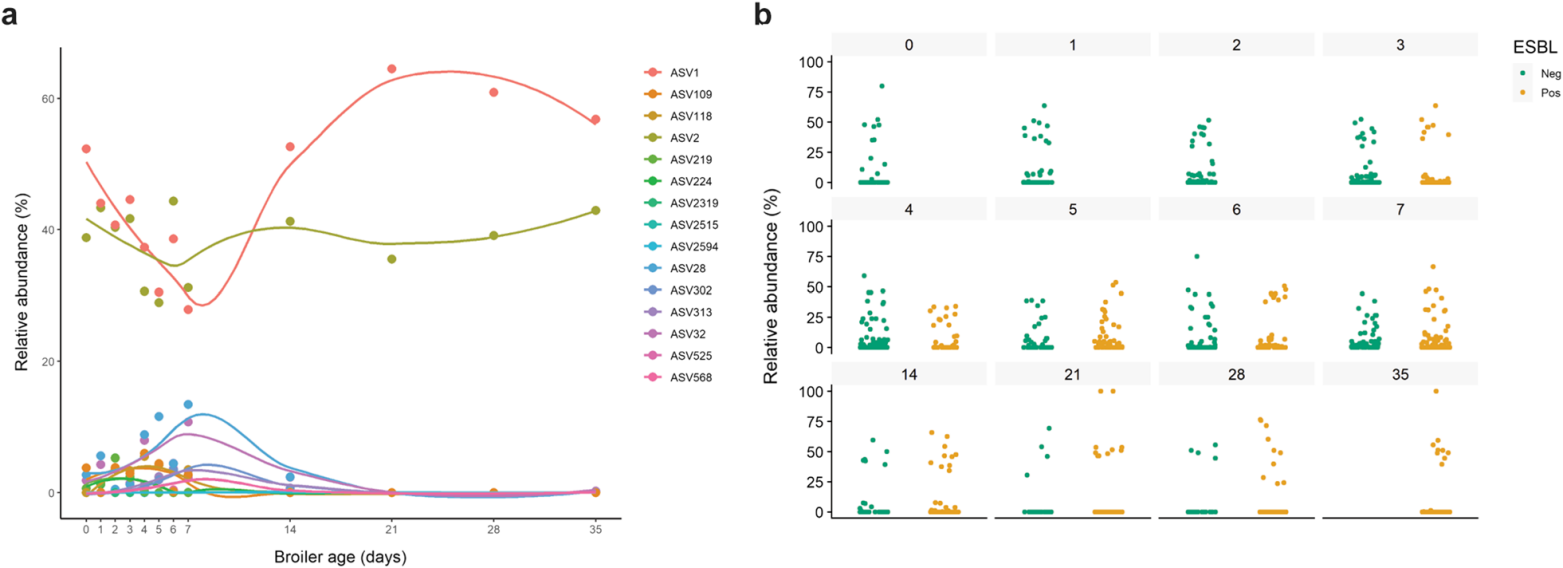
Relative abundance of *Escherichia/Shigella* genus*. ***a** Relative abundance trends of fifteen amplicon sequence variants (ASVs) recovered from the broilers’ caeca and associated with the *Escherichia/Shigella* genus*.* Each dot represents the relative abundance mean of each ASV per time point. **b** *Escherichia/Shigella* relative abundance over time in ESBL-Ec^−^ and ESBL-Ec^+^ broilers. No differential abundance of *Escherichia/Shigella* was observed over time between broilers' groups (ANCOM-BC, *p* > 0.05)

Fifteen amplicon sequence variants (ASVs) associated with *Escherichia/Shigella* were recovered (Fig. 3a), but only two persisted over time. Across all samples, *Escherichia/Shigella* was dominant both in ESBL-Ec^+^ (6.5% ± 7.1) and ESBL-Ec^−^ broilers (17.7% ± 19.3), but they decreased over time, from 37.3% ± 24.0 on day 1, to 1.3% ± 2.2 on day 35. Importantly, on day 1, all chickens were ESBL ESBL-Ec^−^. The relative abundance of *Escherichia/Shigella* decreased as broilers aged. No differential abundance of *Escherichia/Shigella* between ESBL-Ec^+^ and ESBL-Ec^−^ broilers were observed over time (ANCOM-BC, *p* > 0.05; Fig. 3b).

### Alpha diversity (microbial richness and evenness)

**Fig. 4:**
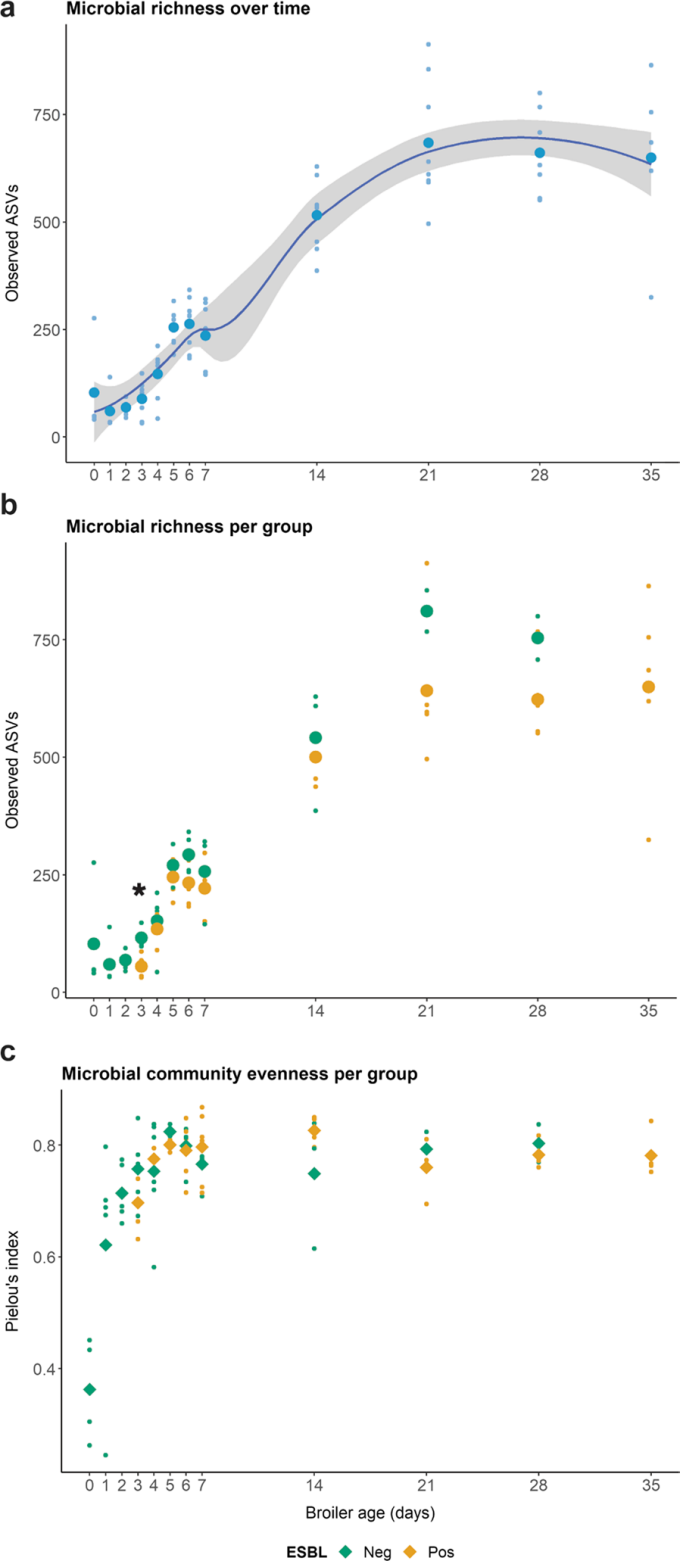
Caecal microbiota richness and evenness of ESBL-Ec^−^ and ESBL-Ec^+^ broilers.** a** Observed microbiota richness in all broilers during their production life cycle. Small dots represent the amplicon sequences variants (ASVs) observed in each individual broiler and enlarged dots represent the ASVs mean from all broilers samples in each time point. The grey area represents the 95% confidence interval. Age significantly predicted microbial richness over time (*p* < 0.001). **b** Comparison of microbiota richness between ESBL-Ec^−^ and ESBL-Ec^+^ broilers. The asterisk denotes a *p* < 0.05 for the Wilcoxon rank-sum test. **c** Comparison of microbiota evenness between ESBL-Ec^+^ and ESBL-Ec^−^ broilers

To study the richness and evenness of the caecal microbiota, we measured the number of observed ASVs and Pielou's evenness, respectively. Microbial richness increased with the broilers' age, from 103.3 ± 115.2 ASVs on day zero to 649.6 ± 202.9 on day 35 (Fig. 4a). Broiler’s age significantly predicted microbial richness over time with the formula: observed richness = 101.24 + 20.87 x (time), *Adj R*^*2*^ = 0.76, *p* < 0.001. Over time, the observed richness was consistently higher in ESBL-Ec^−^ broilers (Fig. 4b), but significant differences between both groups were found exclusively on day three, when ESBL-Ec^−^ showed a higher number of ASVs than ESBL-Ec^+^ broilers (Wilcoxon rank-sum test, *p* = 0.016).

Community evenness rapidly increased in young broilers (day 0–5) and plateaued after five days in both groups (Fig. 4c). No significant differences were found between ESBL-Ec^+^ and ESBL-Ec^−^ broilers (Wilcoxon rank-sum test, *p* > 0.05).

### Beta diversity

**Fig. 5:**
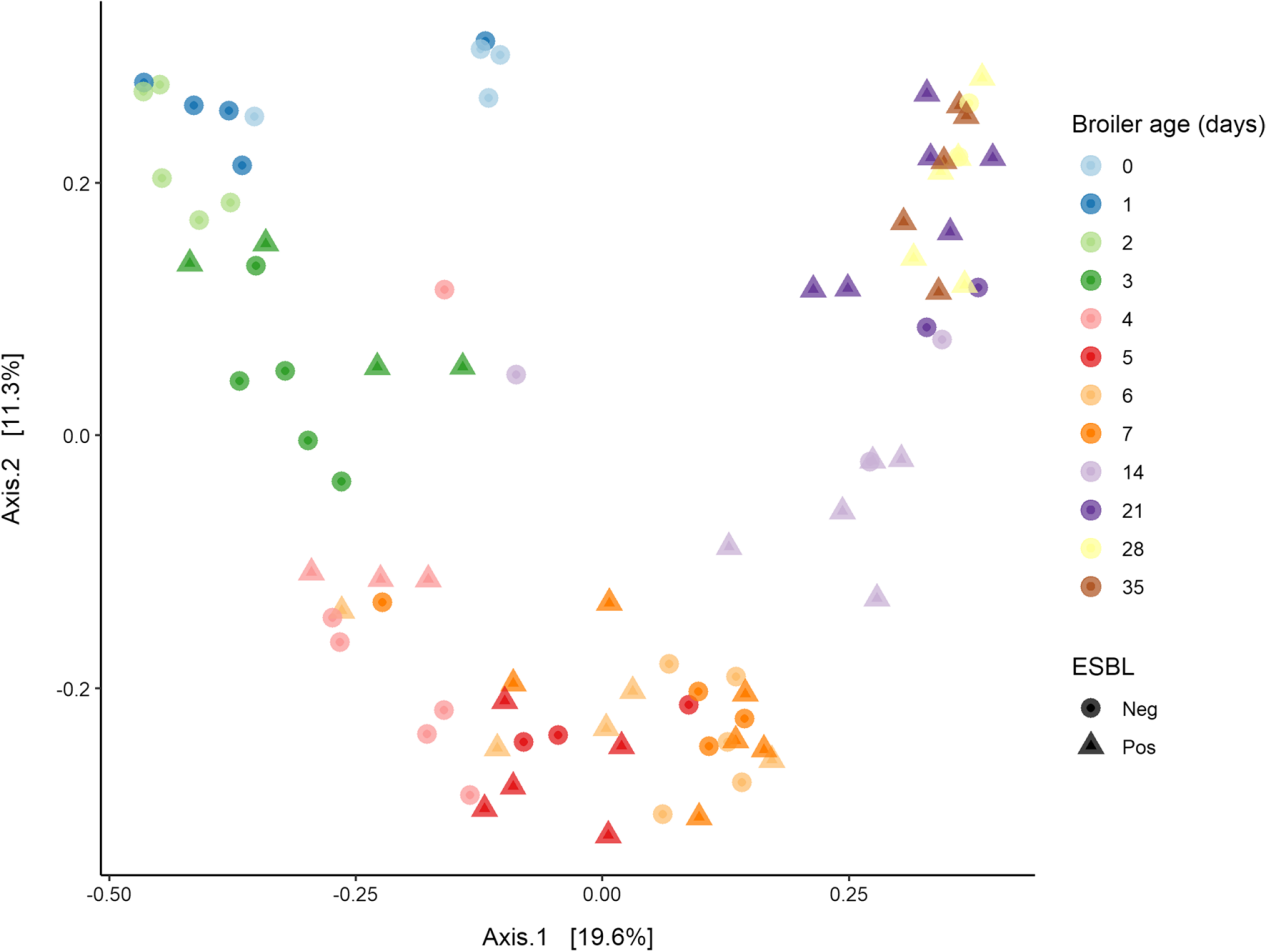
Principal coordinate analysis based on Bray Curtis dissimilarity metrics (BC-PCoA). Changes in the caecal microbial community composition over time (day 0–35). Age explained 14% of the compositional variation of the caecal microbiome (BC-dbRDA: *F* (2,66) = 6.47, *p* = 0.001). ESBL-status explained no variability

Changes in the caecal microbiota composition were analysed with Bray–Curtis principal coordinate (BC-PCoA) and Bray–Curtis distance-based RDA (BC-dbRDA) analyses. Samples clustered significantly according to age (Adonis, *p* < 0.001; Fig. 5). Pairwise analysis showed significant differences between all days pairing (Pairwise Adonis, *p* < 0.05), except for days 0–1 and days 28–35. No significant clustering by ESBL-status was observed in samples from days 3–28. No collinearity was found between age and ESBL (*VIF* < 3). BC-dbRDA showed no explanatory power of ESBL status, while age explained 14% of the compositional variation of the caecal microbiota, *F* (2, 66) = 6.47, *p* = 0.001.

## Discussion

Exploring the successional dynamics of the caecal microbiota of broilers can reveal windows of opportunity to implement intervention strategies that reduce the spread of resistant commensal and pathogenic bacteria. However, little is known about how changes in the developing caecal microbiota affect the prevalence of ESBL-Ec or how the presence of ESBL-Ec affects the microbiota. Here, we studied the caecal microbiota of developing commercial broilers in conventional farming conditions and classified them as ESBL-Ec^+^ and ESBL-Ec^−^. We found no clear divergence between these two groups over time, suggesting that the presence of ESBL-Ec does not have consistent effects on the caecal microbiota of developing broilers.

All broilers tested negative for ESBL-Ec on days zero and one. ESBL-Ec was detected only from day two onwards, and its prevalence increased rapidly over time, suggesting that the farm environment was the likely source for ESBL-Ec colonisation. The AMR phenotype, ESBL-gene, plasmid typing and MLST results were identical for all samples, indicating a clonal spread of ESBL-Ec throughout the flock. The clonal distribution of ESBL-Ec has been associated with the high shedding of ESBL-Ec after colonisation in previous studies [14].

In contrast to the ESBL-Ec prevalence trends, the relative abundance of *Escherichia/Shigella* decreased over time in both groups of broilers. This reflects the broilers' microbiota dynamic development over time; as broilers age, the microbial diversity increases, causing shifts in bacterial abundances. Despite the continuous dominance of members of the phylum *Firmicutes*, *Escherichia/Shigella* persisted throughout the broilers production life cycle. The observed changes in the caecal microbiota composition over time resembled those documented in previous studies: the relative abundance of *Proteobacteria* decreased as broilers aged, while the relative abundance of *Firmicutes* and *Bacteroidetes* gradually increased [16, 18, 19].

A possible explanation for the high prevalence of ESBL-Ec in broilers at day 35, when the relative abundance of *Escherichia/Shigella* was not higher than 5%, is that other microbiota community members might acquire, carry and contribute to the spread of ESBL genes. A previous in vitro study reported the successful transfer of CMY-2-encoding IncI1 plasmids from exogenous *E. coli* to members of the human gut microbiota [20]. IncI1 plasmids have been described as promiscuous plasmids that can migrate within the members of the order *Enterobacterales* and likely between different bacterial species [21, 22]. To better understand the prevalence of ESBL-Ec in the broilers' caecal microbiota, we intend to quantify *E. coli* (16S rRNA gene copy numbers) by quantitative PCR (qPCR) in future studies.

Consistent with the literature [16, 18, 19], the observed caecal microbial richness increased linearly as broilers aged in both groups. Overall, microbial richness was not significantly different between groups. However, a higher microbial richness was consistently observed in ESBL-Ec^−^ compared to ESBL-Ec^+^ broilers over time. In line with this finding, ESBL-colonised broilers showed a much less diverse microbial composition on day three, with only five genera representing 86% of the total community. These results suggest small but consistent alterations in the microbiota's competitive landscape, which could be further explored with controlled, culture-based laboratory experiments.

As shown in previous research [16, 19, 23, 24], host ageing is one of the drivers of microbiota composition. In our study, age explained 14% of the microbial composition variability. The succession of the broilers' microbial communities was observed in three stages. The first stage (days 0–4) was dominated by *Firmicutes* and *Proteobacteria*, mainly by families *Clostridiaceae*, *Enterococcaceae* and *Enterobacteriaceae.* A decrease in *Proteobacteria* (< 10%) and a complete dominance (> 80%) by members of the *Firmicutes* (families *Ruminococcaceae*, *Lachinospiraceae* and *Lactobacillaceae*) characterised the second stage (days 5–14). In the third stage (days 21–35), *Proteobacteria* continued steadily decreasing (< 5%), while *Bacteroidetes* emerged, accounting for 16.6% of the total community. *Firmicutes* were still extensively represented principally by families *Ruminococcaceae* and *Lachinospiraceae* and the appearance of members of the *Clostridiales.* Despite the differences in study designs, these observations support previous results [16, 19], in which successional dynamics were also characterised in 3 stages and represented by similar phyla and families. On the other hand, the colonisation by ESBL-Ec did not explain any variability in the caecal microbiota composition in this study. Our results match previous observations [25], in which asymptomatic gut carriage of ESBL-Ec was not associated with differences in microbiota composition in humans.

To the best of our knowledge, this is the first study assessing the differences in the microbiota composition and diversity of ESBL-Ec^+^ and ESBL-Ec^−^ broilers from a commercial farm throughout the production round. A previous experimental study examined the microbiota of broilers colonised with ESBL-Ec and treated with competitive exclusion products on days 5 and 21 [26]. However, the study aimed to evaluate the effect of compartmentalisation and interventions on the transmission and prevention of ESBL-Ec colonisation in the broiler microbiota composition more than the effect of ESBL-Ec on the broilers caecal microbiota. Longitudinal-experimental studies which manipulate ESBL-Ec prevalence could reveal the process of colonisation of ESBL-Ec in the caecal microbiota and shed light on the relationship between AMR bacteria, the caecal microbiota, and potential avenues for microbiota-based control of AMR. Our study highlights the need to consider the natural dynamics of the host-microbiota development and colonisation by resistant bacteria.

## Conclusions

This study contributes to assessing the role of ESBL-Ec in the successional dynamics of the caecal microbiota in developing broilers and shows that the presence of ESBL-Ec is associated with mild but consistent reductions in alpha diversity and transient microbiota compositional differences. Our study further documents the clonal spread of ESBL-Ec and points at the farm environment as a likely source for ESBLs. We also report the ESBL-Ec prevalence trend in a single broilers’ flock and during a single production round. Future research should aim to determine if the observed patterns of ESBL-Ec spread are common across production cycles and farms and seek to more precisely understand whether the presence of ESBL-Ec modulates the competitive landscape of the broiler microbiota or vice-versa.

## Materials and methods

### Sample collection

Broiler chickens (*Gallus gallus domesticus*) originated from a single flock in a Dutch commercial broiler farm. Fertilised eggs were hatched on the broiler farm. In total, 216 birds were randomly sampled at 12 different time points of the production cycle (days 0–7, 14, 21, 28, and 35 of age), beginning on the day of hatching. Eighteen chickens were euthanised for caecal sample collection at each time point. Samples were collected between October and December 2017. Caecal content was aseptically collected from individual birds. A portion was used for cultivation within 4 h, while the rest of the sample was preserved for further analyses.

### Selective isolation of ESBL-*E. coli*

Selective culture media was used for screening the caecal samples for the presence of ESBL-producing *E. coli*. Samples (n = 216) were aseptically collected from individual caeca by using sterile swabs. The collected swabs were placed on 3 mL of peptone water for bacterial enrichment and incubated overnight at 37 °C. A day after, 10 µL of the enrichment were inoculated on a MacConkey agar plate containing 1 mg/L cefotaxime. After overnight incubation at 44 °C, a suspected *E. coli* colony was randomly selected, sub-cultured on Heart Infusion Sheep blood (5%) agar (HIS) and incubated at 37 °C overnight. Colonies were confirmed as *E. coli* by MALDI-TOF mass spectrometry (Maldi Biotyper Compass, Bruker®).

### Antimicrobial susceptibility testing

Antimicrobial phenotypes were determined by broth microdilution according to ISO 20776–1 using a commercially available antibiotic panel (plate format EUVSEC3, (Thermo Scientific) intended for antimicrobial monitoring of *E. coli* and *Salmonella* according to the Commission Implementing Decision (EU) 2020/1729 based on EFSA guidelines) [27]. Minimum inhibitory concentration (MIC) results were interpreted with published epidemiological cut-off values (ECOFF) from the European Committee on Antimicrobial Susceptibility Testing (EUCAST) [28].

### Plasmid identification

Putative ESBL-*E. coli* isolates were selected for gene and plasmid identification using real-time PCR [29] and PCR Based Replicon Typing [30]. Isolates were grown in Luria-Bertani (LB) broth and incubated overnight at 37 °C (shaking). Plasmid DNA was extracted as previously described [31] and used for bacterial transformation. For this, plasmid DNA (1 μL) was mixed with 12 μL of electrocompetent cells (ElectroMax™ DH10B cells, Gibco Invitrogen). The mix was added to a 0.1 cm gap length cuvette and kept on ice. Electroporation was performed using 200 Ω – 1.25 kV—25 μF. Cells were incubated at 37 °C for 45 min on LB broth and then streaked on LB agar plates containing 1 mg/L cefotaxime. After overnight incubation (37 °C), one putative transformant colony was selected per plate and restreaked on selective LB agar. DNA of putative transformants was extracted for i. resistance gene confirmation and ii. plasmid identification by PCR Based Replicon Typing (PBRT 2.0 Diatheva) according to the manufacturer's recommendations.

### Nucleic acid extraction

DNA was extracted from 0.2 g of caecal content using the Qiagen kit QIAamp Fast DNA stool mini kit (Qiagen, Hilden, Germany). In addition to the manufacturer's protocol, a bead-beating step was included at the beginning of the extraction; samples were treated in a FastPrep-24 5G® at 30 Hz for 30 s in each of the three cycles. DNA was eluted in 35 uL of nuclease-free water. The ZymoBIOMICS Microbial Community Standard (Zymo Research) was used as positive control for the DNA extraction and downstream processing steps.

### Amplification and 16S rRNA gene sequencing

Extracted DNA was quantified using a CLARIOstar® (BMG Labtech, Ortenberg, Germany). The V3-V4 region of the 16S rRNA gene was amplified in all the samples in triplicate to minimise PCR biases. The PCR reaction consisted of 25 μL total volume, including 12.5 μL of Q5 master mix, 1 μL of V3V4 primer mix (CVI_V3-forw 5' CCTACGGGAGGCAGCAG 3' and CVI_V4-rev 5' GGACTACHVGGGTWTCT 3'), 2.5 μL DNA and 9 μL of nuclease-free water. Amplification conditions consisted of 98 °C for 2 min, 20 cycles at 98 °C for 10 s, 55 °C for 30 s, 72 °C for 10 s, and finally 72 °C for 7 min. PCR products were pooled, analysed on a 48 well 2% E-gel (ThermoFisher Scientific) and then sequenced on a Miseq (Illumina, San Diego, CA) using a 2 × 300 bp paired-end cycle sequencing run.

### Amplicon sequence variant (ASV) identification and taxonomy assignment

Analyses of the 16S rRNA gene sequence reads were conducted in R 3.6.3 [32] with the *dada2* v1.14.0 package [33]. Low-quality reads were filtered and trimmed using the parameters truncLenFR = 240,240 and trimLeft = 17, respectively. After merging the forward and reverse reads and removing chimaeras, ASVs were assigned with the SILVA v.132 classifier [34].

### Downstream and statistical analysis

After quality control, 2,582,888 16S rRNA gene sequences were recovered from 89 caecal samples and rarefied to 29,351 reads per sample (rngseed = 1). One sample (day 0) with a low number of reads (< 30) was excluded from the analyses. Alpha diversity (observed richness and evenness) was estimated at the ASV level in all samples using the *phyloseq* v.1.30.0 and *microbiome* v.2.1.26 packages, respectively. Differences in alpha diversity between broiler groups were evaluated by using Wilcoxon rank-sum test with the Benjamini-Hochberg (BH) correction.

Broiler caecal microbiota relative abundance was estimated at phylum, family and genus level with the *phyloseq* v.1.30.0 package. Relative abundances are presented as mean and standard deviations. Differential abundance testing between broilers groups was performed for each time point using the *ANCOMBC* v.1.0.5 package with the Benjamini-Hochberg (BH) correction [35].

Beta diversity (constrain and unconstrained) analyses were performed using the Bray–Curtis distances at the ASV level. Changes in the broiler's caecal microbial community composition were visualised with principal coordinate analysis (PCoA) and tested with permutational multivariate analysis of variance (Adonis). Distance-based redundancy analysis (db-RDA) and variation partitioning were performed to assess the effect of age and ESBL-status on the broiler's microbial caecal composition using the functions *dbrda* and *varPart* from the *vegan *(v.2.5-7) package. dbRDA models were tested for multicollinearity using the variance inflation factors (VIF) with the function *vic.cca* from the *vegan *(v.2.5-7) package [36].

### ESBL-Ec prevalence

Prevalences with their associated 95% confidence intervals based on the 2.5th and 97.5th percentiles were estimated for each time point. Confidence intervals were calculated using the Clopper-Pearson binomial method [37] from the *prevalence *(v.0.4.0) package [38]. Prevalence was calculated, and trends in ESBL-Ec prevalence were tested with a chi-squared test for trends in proportions using the function *prop.trend.test* from the *stats *(v.3.6.2) package.

## Supplementary Information

**Additional file1**. Overview of the broilers’ caecal samples and molecular typing of ESBL producing *E. coli.*

**Additional file2**. Antimicrobial susceptibility testing and phenotype resistance profiles of ESBL producing *E. coli.*

**Additional file3**. Relative abundance of caecal bacterial families observed in ESBL-Ec- and ESBL-Ec+ broilers. No differential abundance between broiler groups were observed over time (ANCOM-BC, p > 0.05).

## Data Availability

The datasets generated and/or analysed in the current study are available in the Figshare repository https://figshare.com/articles/dataset/Data_and_analyses_used_in_the_research_paper_Succession_in_the_caecal_microbiota_of_developing_broilers_colonised_by_Extended-spectrum_-lactamase-producing_Escherichia_coli_/16611091.

## Abbreviations

AMR: Antimicrobial resistance
ANCOM*-*BC: Analysis of compositions of microbiomes with bias correction
ASV: Amplicon sequence variant
BC-dbRDA: Bray Curtis distance-based RDA
BC-PCoA: Bray–Curtis principal coordinate
ECOFF: Epidemiological cut-off values
ESBL-Ec: Extended-spectrum β-lactamase-producing *Escherichia coli*
ESBL-Ec^+^: ESBL-Ec colonised broilers
ESBL-Ec^−^: ESBL-Ec non-colonised broilers
ESC: Extended-spectrum cephalosporins
EUCAST: European Committee on Antimicrobial Susceptibility Testing
HGT: Horizontal gene transfer
MIC: Minimum inhibitory concentration
MLST: Multilocus sequence typing
pMLST: Plasmid multilocus sequence typing
PBRT: Bacterial transformation and subsequent PCR-based replicon typing
