# Supplementary material for: Succession in the caecal microbiota of developing broilers colonised by extended-spectrum β-lactamase-producing Escherichia coli
Source: Anim Microbiome. 2022 Aug 19;4:51. doi: 10.1186/s42523-022-00199-4 (PMC9389726; doi:10.1186/s42523-022-00199-4)
Supplement: Supplementary file 3 — Additional file3. Relative abundance of caecal bacterial families observed in ESBL-Ec- and ESBL-Ec+ broilers. No differential abundance between broiler groups were observed over time (ANCOM-BC, p > 0.05). [file 42523_2022_199_MOESM3_ESM.pdf]

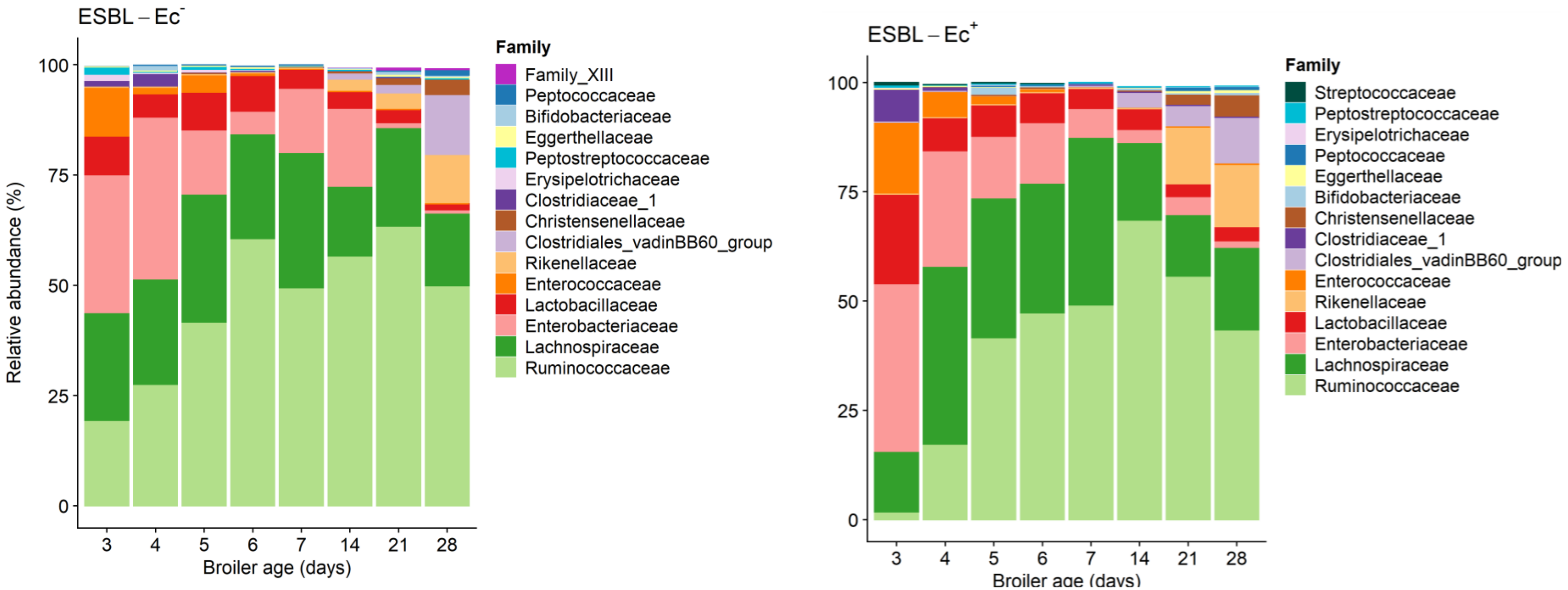

Additional file 3. Relative abundance of caecal bacterial families observed in ESBL-Ec^{-} and ESBL-Ec^{+} broilers from day 3 to 28. The relative abundance of each family is shown in decreasing order from bottom to top.

Succession in the caecal microbiota of developing broilers colonised by extended-spectrum β-lactamase-producing *Escherichia coli*

Ingrid Cárdenas Rey, Teresita d.J. Bello Gonzalez, Jeanet van der Goot, Daniela Ceccarelli, Gerwin Bouwhuis, Danielle Schillemans, Stephanie D. Jurburg, Kees T. Veldman, J. Arjan G.M. de Visser, Michael S. M. Brouwer.
